# Rapport à l'Académic Royale de Médecine sur la Peste et les Quarantaines fait au nom d'une Commission

**Published:** 1847-07

**Authors:** 


### Art. XVII.

1. *Rapport à l'Académie Royale de Médecine sur la Peste et les Quarantaines fait au nom d'une Commission.* Par M. le Dr. Prus. *Accompagné de Pièces et Documents, et suivi de la Discussion dans le sein de l'Académie.—Paris,* 1846. 8vo, pp. 1050.
2. *A Treatise on the Plague, more especially on the Police Management of that Disease, illustrated by the Plan of Operations successfully carried into Effect in the late Plague of Corfu; with Hints on Quarantine.* By A. White, M.D., Deputy Inspector of Military Hospitals, late Superintendent of the Plague in Corfu, &c.—*London,* 1846. 8vo, pp. 342.
3. *Correspondence respecting the Quarantine Laws, since the Correspondence last presented to Parliament. Presented by Command to the House of Commons, in pursuance of their Address of May* 19, 1846.—*London.* Folio, pp. 48.

In October, 1843, and January, 1845, we drew the attention of our readers to the subject of the quarantine laws in their relation to plague. The appearance of the works whose titles we have transcribed, and recent changes in the quarantine regulations of the Mediterranean and English ports, render it advisable to give some account of what has been done since we wrote on this question. It will be well, however, first to give a short recapitulation of the leading views we advanced, and point out how they are affected by the facts and arguments in the works now before us.

In our first article we entered particularly into the inquiry, "How is plague propagated?" and by deductions from facts selected from the works of authors of the most opposite opinions, and particularly from facts observed in 1813, at Malta, proved that the disease is propagated by contagion; that while *actual contact* with persons affected with plague is not necessary for its propagation, still as their exhalations are not injurious at any considerable distance, a moderate dilution with atmospheric air appearing to render them innocuous, *close approach* is necessary—that the question of propagation of plague by clothes or merchandise was still unsettled—that although the exact period of latency of the disease in the human system could not be accurately defined, all credible evidence tended to prove that it never exceeded ten days; and, lastly, that certain unknown terrestrial or atmospheric conditions are necessary for the extension of the disease as an epidemic, just as certain conditions of air, moisture, and temperature are necessary to the process of fermentation, we might have added as perhaps a more apt illustration, or certain qualities of soil and climate to the germination of plants. The necessary conditions of air, moisture, and temperature may be present, but in the absence of the fermenting principle, no fermentation follows, and on the other hand without these conditions the fermenting principle is inert. Just so with vegetation: a field may be in every way fitted for the growth of a crop, the season and climate may be favorable, but unless the seed be sown no harvest is expected; while if the seed be sown in an ungenial soil or climate, or out of its appointed season, it is equally unproductive. It would be just as reasonable to deny the propagation of plants by the germination of their seeds, because each has its peculiar season, soil, and climate, as to deny the propagation of plague by contagion, because its influence has similar limitations.

We gave reasons for believing that the origin of the plague is now invariably in Lower Egypt, and that a proper system of drainage, sepulture, burial of dead animals, improvement in the dwellings and customs of the people, and proper segregation of every case of plague as it arises, would gradually extirpate the disease as they have done in parts of Turkey, Russia, Greece, Asia Minor, Syria, and Barbary, where it was formerly considered indigenous or endemic. We further showed that plague had never been brought to any European lazaretto except in ships coming from places where it was raging at the time of their departure, and argued from this fact that quarantine should only apply to ships arriving from places so circumstanced. In our second article we brought forward other facts strongly confirming these views, and entered upon a question which has since been warmly agitated,—the possibility of eradicating or totally destroying the plague,—and gave our reasons for believing that the seeds of contagion were preserved in Egypt in certain places where sporadic cases occur throughout the year, by the peculiar manner of burying the dead. We showed that the receptacles for the dead, are in all probability nurseries of the disease, and advocated their destruction with the introduction of a new mode of sepulture.

It will be seen that the views we advanced were not those of others. Writers had been previously divided into two classes, contagionists and non-contagionists, the former advocating a continuance of quarantine regulations, the latter their absolute repeal. We, on the other hand, argue that the non-contagionists are the real supporters of these regulations, for it is owing to their advice that the plague is still existing in

Egypt, while the contagionist is the true enemy of useless quarantine laws, reducing them to the limits of utility and real necessity, and labouring to destroy the disease upon which their existence depends. Large extracts from our articles were translated into the journals of Malta, Marseilles, Athens, Smyrna, and Constantinople, and we have since seen that our views have been generally adopted by those journals; further than this, Captain Carolani, a most intelligent health-officer of Malta, the captain of the lazaretto there, previously urged them upon Mehemet Ali in a personal interview. We should be sorry to assume any unmerited credit, yet it is not a little singular that we find Sir W. Pym making the following statement in 1845.

"The towns of Alexandria and Cairo are remarkably clean, and Alexandria in particular is rapidly improving;—orders having been issued for widening streets, pulling down houses, and more particularly the huts of soldiers of the lower order, which are rebuilt upon a much better scale. The health regulations are not confined to the towns, but extend over all Lower Egypt, and particular attention is paid to the Delta, medical men being stationed in every district with most particular instructions for acting against any suspicious case of plague. They immediately separate the sick from the healthy, and cut off communication with the individuals of the family, and keep them in quarantine; they have the power of pulling down houses, calling in the military if necessary to their assistance, and in fact they appear to have declared war against plague, and appear confident that the disease may be eradicated. Mehemet Ali himself seems convinced of it, and when I mentioned to him that there was a very short quarantine in England against Egypt, his reply was, 'There ought to be no quarantine, it is our own fault;' he repeated, 'it is our own fault. We must get rid of the plague.'" (Correspondence, p. 17.)

In May, 1846, the parliamentary correspondence from which we have just quoted, and the report to the French Royal Academy of Medicine, by a committee of eleven of its members, were published. The former consists of correspondence between our Foreign Office and those of France and Austria, with regard to the formation of an European congress to establish a uniform period of quarantine for travellers and merchandise, and an account from Sir W. Pym, of visits made by him in 1844 and 1845 to the different lazarettos of the Mediterranean, Adriatic, and Levant. Nothing has resulted from the proposal for a congress, owing to delay and difficulties on the part of Prince Metternich, and it is not a little amusing to trace the manner in which he puts off from time to time the establishment of regulations which would check the rapidly growing importance of the Austrian port Trieste. Vessels have been for several years past admitted to this port with but nominal quarantine, and the consequence has been an increasing afflux of ships, to the injury of Marseilles and the Italian ports. A uniform period of quarantine would destroy this advantage, and therefore the clever Austrian has very politely thrown dust in the eyes of his diplomatic friends. We have already extracted the only important remark in the letter of Sir W. Pym, but shall hereafter notice a most important document he incloses, showing the periods of latency in all the cases of plague which have occurred in the lazaretto of Alexandria, from 1840 to 1843 inclusive. Some interesting papers are also returned from Malta, giving an account of all vessels which have arrived there with plague on board, since the island has been in the possession of Great Britain, but we have given the facts stated in the returns in our former articles.

Dr. White's work is divided into five parts: a dissertation on the nature and qualities of plague; its origin, history, symptoms, and progress; an account of the plague of Corfu in 1816, and its police management, with general remarks on treatment. Mr. Tully had previously published an account of the same plague, but Dr. White regarding it as defective in the "description of the extension of the calamity from place to place, and in the detail of the police treatment adopted on the occasion," has brought forth the present work, censuring Mr. Tully, with apparent justice, for disingenuously concealing the subordinate situation he held under Dr. White during the whole progress of the disease, and assuming the credit of originating measures which he merely carried out under the direction of his superior. The value of the work mainly depends upon the account of the police management of the plague of Corfu, which we think might have been usefully published alone in a smaller and less expensive form, as the remainder, although well written, is a rather common-place compilation from well-known sources of information, and deficient in exact accounts, which the author might easily have obtained, of cases of importation into lazarettos.

The report to the French Academy consists of three parts, the report of the committee, returns and documents in support of their conclusions, and the particular views of individual members, as expressed by them in the general academic discussions. The report states that the committee has especially studied the plagues "of Nimeguen in 1635, of London in 1665, of Marseilles in 1720, of Transylvania, in 1755, of Moscow in 1771, and of Egypt in 1798-99, and 1800." (p. 3.)

It is rather singular that the plagues of Malta in 1813, and Corfu in 1816, of which Hennen, Faulkner, and Tully had published ample accounts—plagues observed in our own time in countries where local origin could not be suspected—should have escaped the notice of the committee, while they have studied those which occurred two centuries since, over the accounts of which much more doubt must necessarily hang. The studies of the French physicians in Egypt since 1835; the work of Dr. Brayer, entitled 'Nine Years in Constantinople;' Dr. Gosse's account of the plague in Greece in 1828-29; Drs. Morea and Hemso on the plagues of Noja and Morocco in 1817-18; original manuscripts regarding all cases of plague observed in the lazaretto of Marseilles since 1720; with numerous despatches from the French consuls and ambassadors in the Levant, with other private communications, have furnished the body of evidence upon which the committee has founded the report. It is divided into four parts.

"In the *first part* we shall inquire in what countries the plague has been known to develop itself spontaneously; we shall then endeavour to determine the causes of the spontaneous plague; we shall show that when these causes have ceased to exist in Egypt and elsewhere, the plague has disappeared; we indicate the countries where the persistence of these causes renders the plague endemic, or, at least, makes us fear the return of the spontaneous plague; lastly, we insist upon the true prophylactic measures of the spontaneous plague.

"In the *second part* we shall answer the three following questions:

"1. Has the plague always shown itself with the principal characters of epidemic diseases, when it has broken out with violence in Africa, Asia, and Europe?

"2. What are the differential characters of the epidemic and sporadic plague?

"3. Is the plague propagated like epidemic diseases, that is to say, by the migration of certain atmospheric influences, and independently of the action that impested persons can exert?

"In the *third part* we shall consider the transmissibility of the plague: Is it transmissible by inoculation? Is it transmissible in and beyond epidemic foci, by the immediate contact of the impested, by the contact of goods, clothing, or merchandise, or by the miasmata exhaled by the impested, of which the air is the vehicle? We shall conclude the third part by the examination of the three following questions:

"1. Can persons affected with sporadic plague give rise to foci of infection sufficiently active to transmit the disease?

"2. Is the plague more or less transmissible according to the intensity of the epidemic, in relation with its first, second, or third periods, or with the organic dispositions of the individuals submitted to the action of the pestilential miasmata?

"In the *fourth and last part* we shall inquire what is the ordinary and exceptional duration of the incubation of plague.

"Then follow the conclusions of our report, and the applications of these conclusions to the questions of quarantine." (pp. 10-11.)

We shall follow the course of the report, giving a condensed statement of the most important results of the inquiries of the committee, with such comments as may appear necessary or desirable.

The following definition of the plague appears to be unobjectionable:

"The plague is a disease of the whole organism, in which the nervous, sanguineous, and lymphatic systems are especially affected, and which is generally characterized exteriorly by buboes, carbuncles, and petechiæ." (p. 11.)

With regard to the countries where the plague arises spontaneously, the committee clearly prove by a quotation only recently discovered in the work of Rufus of Ephesus, a physician who lived under Trajan, that the plague, such as is seen at present, incontestably existed in Egypt at least two centuries before Jesus Christ, the country which all modern research points out as its native seat. They go on to argue that it also arises spontaneously on the banks of the Danube, as well as those of the Nile and Euphrates, and their general conclusion is that "the plague has been known to arise spontaneously, not only in Egypt, Syria, and Turkey, but also in many other countries of Africa, Asia, and Europe." (p. 21.)

We dissent altogether from this conclusion, and can find nothing in any of the documents brought forward by the committee to warrant its acceptance. It is in direct opposition to the fact, that since the establishment of quarantine regulations in Europe, Barbary, and Greece, there has been no plague, the importation of which from Egypt has not been distinctly traced, and scarcely in accordance with the paragraph immediately preceding the conclusion: "at the present moment it is therefore almost exclusively from Egypt that we have to fear the importation of the plague." (p. 21.)

The only arguments at all favoring the conclusion are, that it has existed without proofs of importation near the Danube and in Turkey; but on examining the documents it appears far from certain that the epidemic on the banks of the Danube is really plague, or anything more than a severe remittent marsh fever; and the sanitary committee of Constantinople have satisfactorily proved that cases apparently spontaneous in Turkey, have really resulted from infractions of the quarantine regulations, and are among the strongest proofs of the universal dependence of the disease in Turkey upon importation from Egypt. Giving every latitude to the facts brought forward by the committee, they can prove nothing more than *probability*, and therefore the conclusion should have been, that it is not yet determined whether the disease arises spontaneously in Egypt only, although our own firm opinion is, that this country is its sole original seat.

The next question is, whether in the countries where the plague spontaneously arises hygienic conditions exist to which its development may be rationally attributed; and after a most interesting account of the state of the people, their habits and habitations in Egypt, Constantinople, Armenia, and the banks of the Danube, this conclusion is arrived at:

"In all the countries where spontaneous plague has been observed, its development may have been rationally attributed to determinate conditions, acting upon a great part of the population. These conditions are especially: habitation upon alluvial or marshy soil near the Mediterranean Sea or the rivers Nile, Euphrates, and Danube; low, ill-ventilated, crowded dwellings; warm and moist atmosphere; the action of animal and vegetable matters in a state of putrefaction; unwholesome and insufficient food; great physical and moral distress." (p. 35.)

Yet in other countries there are marshy sea-shores, and great rivers with a large extent of alluvial soil continually deposited, and marshes where animals, insects, and vegetables putrefy under a burning sun, and we hear of intermittent and remittent disease as the result but not the plague. In other countries poverty, filth, and famine decimate our race, but not by the plague. All the hygienic conditions enumerated in the conclusion are doubtless predisposing causes of disease, and causes which lead to the devastating spread of the plague when it has once appeared, but we do not see any reason why *alone* they should rather produce plague than smallpox, scarlatina, yellow fever, or any other specific disease. The seed must be sown before the harvest can be looked for, and we shall hereafter show that the committee have scarcely alluded to the real cause of the continued existence of the disease in Egypt.

The next conclusion is that the plague is now endemic in Lower Egypt, and exists there every year in the sporadic form, and about every ten years as an epidemic, and that "the absence in Egypt of any pestilential epidemic during the long period that a good administration and sanitary police in the country victoriously combated the productive causes of the plague, justifies the hope that the employment of the same means would be followed by the same results." (p. 40.)

Now the statement of the committee as to the epidemic periods is contradicted by official reports furnished to and published by them, by which it appears that from 1700 to 1838, that is, during a period of 138 years, the plague has appeared in Egypt as an epidemic seventeen times in successive years, nine times at an interval of two years, twice of three years, once of four, six of five, twice of six, twice of seven, twice of eight, once of nine, and only twice of ten. So much for the accuracy of the deductions of the committee from the facts before them.

The question is of extreme interest, why Egypt, described by Herodotus as the most healthy country of the world, a country which was free from pestilential epidemics during 194 years' occupation by the Persians, during 301 years under Alexander and the Ptolemies, and during a great part of the Roman domination, which commenced 30 B. C. and continued until

620 A. D., has since the commencement of the Arabic rule been so often decimated by the plague. The statement of Rufus before alluded to, although proving that the disease was known, also proves that it was nothing more than a sporadic disease in his time, and a casual allusion only of Galen, who was of the school of Alexandria, would prove the same at a like period. The committee state that Alexandria, which was founded 331 B. C. was, *according to Galen,* attacked by plague for the first time as a pestilence A. D. 263. But they have made a strange mistake in chronology, for Galen was born 131 A. D., and if he spoke of the plague of 263, he must have written when he was 132 years of age. The fact is, it is Eusebius, an author by no means noted for accuracy, who describes the epidemic of 263, and the disease he describes appears to have been simply a contagious typhus; Galen, as we have said, only incidentally alludes to plague, while Celsus, Praxagorus, Serapion, Soranus, and above all Cælius Aurelianus, who lived in the fifth century, and practised in Numidia, have been quite silent with regard to any epidemic disease accompanied by buboes or carbuncles. It must therefore have been a rare disease until the great plague of 542, in the time of Justinian, broke out, which we know was regarded by contemporary writers as a new disease.

The ancient salubrity of Egypt must doubtless be ascribed in a great degree to the general prosperity of the people, the canals of Sesostris, the elevation of the towns upon artificial mounds, but we believe, above all, to the practice of embalmment. Ancient Egypt, the mother of the sciences, had recognised the effect of the periodical fertilizing inundations of the Nile, and of the burning heat of the sun upon the deposit left on the subsidence of the waters of the river, over spots where men or animals were buried. What was the result? Inhumation was forbidden, embalmment enjoined, and now, instead of tombs and cemeteries the traveller observes, along the ranges of hills which border the Nile immense subterranean cavities miles in extent, which are filled with embalmed organic remains. The living was thus protected from the dead, and to ensure the observance of the laws religious influence was called in to the support of human wisdom,—the law became a religious rite; the influence of the divinity was employed to protect Egypt from the evils of her physical formation. The salubrity of the country ceased with the practice of embalmment. The Christian missionaries proscribed the ancient usages as idolatrous and sinful, and the mode of sepulture gradually fell into disuse, and was finally prohibited and abolished 356 A. D. We have seen that the plague, though before not unknown, was a rare disease; but in 542 sprung up the terrible plague which devastated Egypt, Turkey, and Europe, to the borders of the Atlantic, and, according to Gibbon, destroyed a *hundred millions of people.*

In our former article we gave our reasons for believing that the emanations from the dead bodies buried in Lower Egypt are the real cause of the persistence of the disease in that country—that the disease is sown and preserved by the mode of sepulture—that the living are poisoned by the emanations of the dead. A porous level soil filled with dead bodies, penetrated universally by moisture during the overflowing of the Nile, is, after the subsidence of the waters, heated by a burning sun, and a vast cemetery, in the language of M. Pariset, is converted into a "true distillery of dead bodies." Hear what he says of Cairo—

"This great city is traversed by a canal of stagnant water, and the half is built upon burial-places, in general very superficial; of thirty-five cemeteries which appertain to it, twenty-five occupy, or lately occupied, the interior, so close to the inhabited houses, that the dead appear to form part of the living." (pp. 937.)

Sometimes individual instances are more striking than general statements, we therefore cite the following facts adduced by the same speaker:

"In the winter 1823-4, the Pacha built a cotton-factory at Kelioub, a small town four leagues north of Cairo. The foundations of this factory were laid among ancient and recent tombs. One day, about noon, a mason complained of headache. He was sent home, and at four o'clock he was dead. He was not visited, but eight persons who composed his family died the same evening, with both buboes and carbuncles. Kelioub was soon infected, and, of 5000 inhabitants, lost 2000.........This year there had been a great inundation and violent rains; the plague did not previously exist in the neighbourhood, and Kelioub had received nothing from the exterior.........One, two, or three workmen in this factory were afterwards daily affected.........The second fact is quite recent. All the habitations of the Copts have domestic sepultures. Lately a Copt rebuilt his house, and, on arriving at the burial-place, the workmen to the number of four took the plague." (pp. 938-9.)

If we inquire into the nature of the principle, the living man receives from the dead, the reply must be that as yet chemistry has not resolved the problem; but M. Danger, in his recent researches upon the decomposition of animal matters, and particularly of the earth of burial-grounds, has thrown much light upon the subject. We continue to quote M. Pariset.

"The immediate products of putrid decomposition are not, as has been supposed, elementary bodies, as hydrogen, carbon, and nitrogen, but, on the contrary, vapours, the organized condensible molecules of which are capable of being dried, and being indefinitely preserved in this state, and also of being completely disorganized by a certain combination of heat and moisture. Drawn along (*entrainées*) by gases, aqueous vapours, currents of air and dust, dispersed without being destroyed by pure water, as Desgenettes believed, expelled by sulphuric acid, they are only neutralized by the powerful alkaline bases, lime, potass, soda, ammonia, cinders of vegetables. They are condensed, on the contrary, and not decomposed, by porous bodies, among which fine sand may be classed. While they preserve their peculiar organization they can serve as a ferment, and consequently act upon living beings to disorganize them, finding in them the degree of heat and humidity which ought to destroy them; they are not destroyed, or do not undergo their metamorphoses but by making the being who has received them a partaker, whose life is thus compromised, and sometimes destroyed. We thus understand why, amidst so many injurious agents, the plague is not so injurious to Egypt as it might be. The air is always in motion. It is swept by torrents of dust in all seasons. The germs of the plague, thus dispersed by the winds, are less connected and less active. In the second place, the water of the Nile is very alkaline; spread over the land, it reaches the dead, and temporarily neutralizes the miasmata. But their source is not exhausted. In the mild and moist heat of winter, putrid decomposition recommences. The subtile ferments it produces are condensed in the sandy soil, of which Egypt is formeds and in which the dead repose. When the elevation of the temperature make, the ferments evaporate, man receives them by the superficial absorbent vessels, or by those of the lungs in the act of respiration." (pp. 940-1.)

We have before shown that in some parts of Egypt, to avoid the access of water, the bodies are buried above ground, and these receptacles become still more direct sources of contagion. To destroy the plague, both systems of burial must be changed. The cemeteries must be outside the walls of the cities, and as alkaline substances have the property of neutralizing the miasmata or pernicious exhalations, advantage must be taken of the means Providence has placed in the reach of the people. Egypt is supplied with soda in abundance, lime can be easily procured, and instead of enveloping the bodies in soda, as in ancient times after embalmment, let them be buried surrounded by it, or by lime. Let these alkalies be used as manure, and with cleanliness in the towns and villages, and better food for the people, we may hope to see the country restored to its former state of salubrity. The European powers have shamefully neglected this great question. When the government of a petty province is disputed, the four great powers are not slow to interfere; but when it is proposed to exterminate a disease which has destroyed millions of one species, an Eastern pacha is left without assistance. Yet it would be a noble work not only to afford him advice but effective aid, and instead of employing their forces and exhausting their means to destroy man, to turn them to the purpose of reciprocal preservation.

We must leave, however, this tempting theme, and pass on to the next conclusion of the committee, from which we differ entirely.

"The state of Syria, of Turkey in Europe, and Asia, of the regency of Tripoli, of that of Tunis, and of the empire of Morocco, being nearly the same as at the time when epidemics of plague have arisen spontaneously, nothing authorizes us to believe that similar epidemics cannot again break out there." (p. 43.)

Now it has never been proved that the plague has ever been spontaneously developed in these countries. On the contrary, in all the visitations of which correct information has been handed down to us, proofs of importation are distinct and incontrovertible; and since the establishment of quarantine regulations, not a case of plague has appeared in these countries, except in their lazarettos, then of course imported. We always place some reliance on the general belief of the common people, drawn as it must be from experience, and having had frequent opportunities of conversing with natives of all these countries, we may say that we never met with one who, on being questioned as to where the plague came from, had not some story of importation at hand, while an Egyptian has but two answers to the same question, the "will of God," or "it comes from the earth." We still retain the opinion we long since expressed, that, provided the countries in question properly guard against the importation of plague, any restrictions upon commerce or communication with them are needless and injurious. The committee take this view of Algiers, because they say the habits of the Arabs and Kabyles, and the sanitary regulations enforced by the French, are a sufficient guarantee against the spontaneous development of pestilential diseases. Indeed, Algiers is not subjected to quarantine, although Morocco and the Barbary States, which are in unrestricted communication with Algiers, are so. This absurd inconsistency, which we before pointed out, remains unaltered.

The conclusion of the first part of the report is the following.

"The progress of civilization, and a general and constant application of the laws of hygiene, can alone furnish the means of preventing the development of the spontaneous plague." (p. 48.)

The second part of the report is commenced with an inquiry, whether plague, when it spreads with rapidity, is distinguished by the characters of other epidemic diseases, and these characters are defined as follows.

"A disease is epidemic when in a given time it attacks a great number of persons.

"Epidemic diseases present characters which distinguish them from diseases non-epidemic.

"1. They have in their progress a special course. They have generally three periods: periods of commencement, persistence, and decline or termination. These three periods often do not present either the same symptoms, the same lesions, or the same severity.

"2. During the reign of an epidemic disease, other diseases are less numerous and receive the impress of the dominant affection.

"3. When an epidemic disease rages, it is rare that persons who remain in good health do not, more or less, experience the general influence.

"4. Epidemic diseases frequently return and cease in the same season, and in general have the same duration.

"5. An epidemic disease is frequently preceded by other affections, more or less grave, more or less general, which in a manner serve as forerunners." (pp. 48-9.)

Taking these characters as the test of epidemic diseases, the committee conclude, from the evidence before them, that—

"When the plague has raged with violence in Africa, Asia, and Europe, it has always shown itself with the principal characters of epidemic diseases." (p. 73.)

In this we concur, but would remark that smallpox and the contagious exanthemata have the same distinguishing pecularities when they become prevalent. The committee bring forward some interesting facts to show that the situation of a place, and the condition of its inhabitants, have more to do with the epidemic propagation of plague than communication with other infected places. We think, however, that the statement that "infected persons have left infected places, and have lived or died in localities where the epidemic influence did not reign, without the propagation of the malady," only shows what we before urged, that certain conditions of the air prevent the extension of the disease as an epidemic, and that it requires a considerable condensation of the exhalations from plague patients to render them noxious. One of the facts brought forward by the committee is a most glaring misstatement, completely contradicting the real facts of the case. They say—

"In 1813 the English general, Maitland, governor of Malta, not being able, notwithstanding the most severe measures, to extinguish the plague which reigned in Valletta, resolved to have barracks constructed out of the town, and obliged the inhabitants to go and inhabit them. From this moment the plague completely ceased." (p. 71.)

Now all these statements are absolutely unfounded. The plague was not confined to Valletta, but was scattered over nearly the whole island—it was shut up in one village, some months after Valletta was free,—no barracks were even erected outside the town, and the population never left the city. It was on the contrary, by the strict segregation of infected persons in the town, and not by removal from any particular locality that the plague was stopped. It is singular that the only allusion made by the committee to the plague of Malta, should consist of so important an error; perhaps if our personal knowledge extended to other facts brought forward, we might be able to explain some apparent contradictions in their relations. In this case the mistake is unpardonable, as the works of Hennen, Faulkner, and the dispatches of Sir T. Maitland have made the real facts notorious.

In considering the causes of immunity in certain districts, the *susceptibility* of the population has been too much overlooked, not only with regard to plague but to other epidemic, and contagious diseases. Thus, after a wide-spreading epidemic great numbers of the population are protected by one attack against a second, as a general rule, and a new generation must spring up to supply unprotected victims, in other words, to form a susceptible population. Again, the different varieties of the human race are susceptible in very different degrees. Thus, the Negro enjoys an almost entire immunity from the fevers which are so destructive to the white race on the western coast of Africa, and is peculiarly susceptible to the scourge which devastates the north-eastern. Dr. Aubert-Roche has recorded the proportional mortality of different races during the great plague of Alexandria, in 1835. We subjoin his table with the remark that the Negroes, Nubians, and Arabs lived almost in the same hygienic conditions, all being in free pratique, while, with regard to the remainder of the population, the conclusion must be less certain, as the conditions of isolation and hygiene are not the same for all.

| | | | | |
|---|---|---|---|---|
| The Negroes and Nubians lost . . | 84 | per cent., or | 1528 | in 1800 |
| The Maltese . . . . | 61 | | 367 | 600 |
| Arabs, not soldiers . . . | 55 | | 10,936 | 20,000 |
| Greeks . . . . | 14 | | 257 | 1800 |
| Jews, Armenians, and Copts . . . | 12 | | 482 | 4000 |
| Turks . . . . | 11 | | 678 | 6000 |
| Italians, and other inhabitants of Southern Europe | 7 | | 118 | 1600 |
| French, English, Russians, and Germans . | 5 | | 52 | 1000 |

(p. 67.)

The committee go on to inquire why the sporadic plague, observed each year in Egypt, is regarded as non-contagious, while the epidemic plague is highly so. They say that this is not surprising to the physician who knows that sporadic dysentery is not contagious, while epidemic dysentery is so to a high degree. We never knew that this had been proved, indeed the experience of English observers in the East, would lead to the opposite opinion. The explanation, however, is not at all difficult. The conditions of air, moisture, and temperature of the population in general, or of the impested in particular, may not be such as is required for the transmission of the disease. We know that typhus does not spread while a patient is scattered here and there over the wards of a large hospital, but collect a number in a confined or ill-ventilated space, their emanations are concentrated, and those who inhale them often fall victims. Just so a single plague patient though really exhaling noxious vapours can only exert a feeble and limited action, while if circumstances have exposed several others to the same cause, or have been such as to prevent ventilation of his dwelling, and expose others to his concentrated effluvia, the injurious action is increased by the accession of every victim in unknown proportion. Bearing this in mind we can easily understand the following conclusion:

"The sporadic differs from the epidemic plague, not only by the small number of persons affected by the disease, but also and especially because it does not present the characters belonging to epidemic affections." (p. 75.)

A very important question follows, whether the plague can be propagated in the same manner as most epidemic diseases, that is to say, by the migration of certain atmospheric influences, and independently of the action that may be exercised by impested persons.

"Nothing could be more important, when a pestilential epidemic has reigned in a town, than to know how many sick owe their affection to the epidemic constitution, and how many either to absorption of the miasmata escaped from the impested, or to direct or indirect contact with the latter.

"This study was made for the first time in Egypt in 1835. Dr. Lachèze found that at Alexandria and Cairo the epidemic influence had affected persons well isolated, so as to destroy one individual in four hundred, while the plague destroyed one in three among the population in free pratique, that is to say, exposed at the same time to the action of the epidemic constitution, to that of the miasmata which might escape from the impested, and lastly, to direct or indirect contact with infected persons." (p. 77.)

Clot Bey and his sect account for these facts by the superior social condition and domestic comfort of those who observe quarantine, but this surely cannot explain exemption almost total. It would, doubtless, greatly diminish susceptibility, but not afford such complete immunity from atmospheric influence. In the arsenal at Alexandria during the epidemic of 1835, of 6000 workmen, 300 only were attacked, or 1 in 20, while the proportion in the population in free communication with each other was 1 in 3, and there is not that difference between the social condition of the arsenal labourers and the general population, as between the latter who do and do not isolate themselves. The committee attribute the 300 attacks to the atmospheric influence, as they say no accumulation of pestilential miasmata ever existed, and actual contact could not be considered as a cause of extension. On this last point, however, others are at issue with the committee, and trace all the attacks to disregard or infraction of the regulations of segregation. The conclusion is that—

"The plague, abstracting the influence which the impested can exert, is propagated in the mode of most epidemic diseases, that is to say, by the action of general causes." (p. 79.)

We should rather this were worded as not yet fully proved, and a subject for further investigation, which it certainly is.

The *third part* of the report commences with an account of the results of experiments by inoculation. We need only give the conclusion, which perfectly accords with that formerly stated by ourselves, although at page 738 of the discussion M. Hamont relates some very striking experiments, but unfortunately they were all made during the existence of an epidemic.

"The inoculation of blood drawn from the vein of a plague patient, or of pus from a pestilential bubo, have only furnished equivocal results; the inoculation of the serosity taken from the phlyctenæ of a pestilential carbuncle has never given the plague; therefore it is not proved that the plague can be transmitted by inoculation even under the influence of a pestilential constitution.

"We know of no experiment on the same subject made beyond an epidemic focus." (p. 86.)

After a very full inquiry whether the plague is transmissible by contact of the sick *in places where it is epidemic*, the committee experience difficulty in determining whether in any given case the disease is owing to contact, or to the epidemic or endemic conditions in operation; they therefore conclude as follows:

"An attentive and severe examination of recorded facts establishes on the one hand that the immediate contact of thousands of plague patients has proved without danger to those who have been exposed to it in the open air or in well-ventilated situations, and on the other hand that no rigorous observation demonstrates the transmissibility of the plague by sole contact with the sick." (p. 103.)

Thus they agree with the suggestion we long since offered, that the only unobjectionable observations on the transmission of plague are those collected in districts where the endemic or epidemic causes are not in operation, in countries where the disease does not arise spontaneously. It was on this ground that we attached so much importance to the facts observed in Malta, and we still think that, with those recorded by Dr. White as the result of his experience in Corfu, they afford the most exact body of evidence which has ever been applied to this part of the question.

The conclusion with regard to the contact of the clothes or effects of plague patients is that—

"Facts, in a very great number, prove that the goods and clothing which have served the impested have not communicated the plague to persons who have used them without any previous purification.

"The facts which appear to have given an opposite result can only acquire value if confirmed by new observations made beyond epidemic foci, far from the centres of miasmatic infection, far from the countries where the plague is endemic." (p. 113.)

It must be borne in mind, however, first, that the clothes and effects of persons dead of plague are never sold or used in Egypt until after St. John's Day, that is, until after the season in which plague ceases, in other words, until the degree of heat and moisture in the atmosphere, or some unknown condition, is incompatible with the spread of the disease; 2d, that they are freely exposed to the air. It would be difficult to disprove the opinion of many Egyptian physicians, that when the proper season returns these very clothes, if not well purified by exposure to air, are one great cause of the continual existence of the plague in Egypt.

We fully concur in the next conclusion—

"The transmissibility of the plague by merchandise in the countries where the disease is endemic or epidemic, is not an established fact." (p. 115.)

The succeeding question is whether in *epidemic foci* the plague is transmitted by means of air charged with pestilential miasmata exhaled from the lungs and bodies of the sick, and it is a very difficult one to answer, as the doubt always arises whether fresh cases are owing to epidemic influence or to infection. We therefore extract some important facts which tend to its elucidation.

"In 1835 most of the apothecaries of the plague hospital called Ras-el-Tin, at Alexandria, were Italians, who carried to the extreme the fear of every suspicious contact, and the precautions which they regarded as necessary to preserve them from the scourge. Nevertheless, the service they are obliged to render in the wards, where a great number of plague cases were collected, sufficed to make every one of them contract a mortal plague." (p. 117.)

"In the hospitals of Cairo during the same epidemic of 1835, physicians, apothecaries, and nurses were affected with the plague. Of twenty pupils who were sent there from the medical school of Abouzabel, nineteen died.

"Here again we must inquire if these cases were due to the epidemic influence, to contact or infection?

"We do not think we can answer this question better than by repeating what occurred at Abouzabel when this place was invaded by the pestilential contagion.

"At Abouzabel the plague patients were examined, touched, and treated in elevated and well-ventilated country barracks. Neither the physicians nor the apothecaries were affected, notwithstanding repeated contact with impested men and goods, notwithstanding residence in the epidemic focus. But a free ventilation had prevented the formation of any focus of infection." (pp. 117-8.)

Many other facts of great interest might be quoted, but all are more open than those we have just extracted to the objection of epidemic influence rather than that of infection, and show more strongly than ever that it is in places far from the action of the former, at sea and in the lazarettos of Europe, that the question must be determined. The conclusion is given as follows:

"In epidemic foci the plague is transmissible by the miasmata which the impested exhale, and by the foci of infection which may result from them." (p. 132.)

We now pass to the grand question, "Is the plague transmissible beyond epidemic foci?" An affirmative answer maintains, a negative abolishes quarantine regulations; but as we have already entered so fully into this part of the subject in our former articles, we need do little more than give the conclusion of the committee drawn from authentic notices of 25 ships, which since 1720 have arrived in France and Italy with the plague on board.

"The plague is not only transmitted on board ship among persons leaving the same shores, breathing the same air, having the same exercise, the same habits, the same food. The plague patient deposited in an European lazaretto, becomes the cause which develops in others the affection from which he is suffering." (p. 163.)

It is stated that other facts observed at Venice, Leghorn, and Genoa prove the justice of the conclusions drawn from facts observed at Marseilles, and the general conclusion is the following:

"It is incontestable that the plague is transmissible beyond epidemic foci, both in ships at sea and in the lazarettos of Europe." (p. 165.)

If anything is still required to strengthen this conclusion, nothing could do so more forcibly than the return from the records of the quarantine department of Malta of the vessels which had arrived with the plague on board since 1819, published in the parliamentary correspondence before us. It is singular the committee should not have noticed these facts, as the French consul at Malta appears to have been supplied with a copy in 1845, for the express use of the Academy.

With regard to the mode of transmission, it is concluded that transmission by simple contact cannot be proved. This, however, is a very unnecessary refinement, as it would be difficult to be in such close relation with a person as to touch him without inhaling some of his pulmonary exhalations. As to the danger of transmission beyond epidemic foci by effects and clothes of plague patients, the committee do not deny the danger, but doubt, and think further researches necessary in localities where it is certain the plague is not endemic.

We have already given our opinion of the following statements:

"Nothing proves that merchandise can transport the plague beyond epidemic foci." (p. 170.)

"The classification admitted in our lazarettos of susceptible and non-susceptible objects is not founded upon any fact or experiment worthy of confidence." (p. 175.)

"We will not further prolong these considerations on the means of destroying the pestilential principle. They would be certainly insufficient if it were demonstrated that this principle could remain inherent in goods, clothes, or merchandise, and become the cause of the transmission of the disease. They are and will be completely without object until it shall be proved that the virus or pestilential miasm can be presented in these different receptacles.

"The study of the means of disinfecting goods, clothes, and merchandise is still to be made.

"To be rational, researches on this subject ought to be preceded by proof that these different objects can really become charged with the principle of the plague." (p. 179.)

"The plague may be transmitted beyond epidemic foci by miasmatic infection, that is to say, by air charged with miasmata exhaled from the bodies of the impested.

"We shall follow this conclusion by three others, which are but corollaries, but which it appears useful to detail separately, as upon each of them quarantine regulations are based.

"1. It results from facts detailed in the chapters relative to the transmissibility of the plague in and beyond epidemic foci, that the impested, by vitiating the air of the localities in which they are confined, can create centres of pestilential infection capable of transmitting the disease.

"2. The centres of pestilential infection can persist after the removal of the impested.

"3. The centres of pestilential infection once formed on board a ship, by the presence of one or many sick, may be transported even to great distances." (p. 181.)

The two latter conclusions are drawn from a number of curious facts, and it becomes an interesting question how the virus is preserved, whether by imperceptible incrustations forming on the walls of houses or timbers of of ships, or by other unnoticed means. At a late meeting of the Chemical Society, Dr. R. A. Smith read an account of experiments to determine what was the real evil in the polluted atmosphere of towns, and stated that the moisture condensed from the breath contained organic matter in large quantities; and when collected from the windows of crowded rooms, it smelt strongly of human sweat during the evaporation and when the solid residue was heated, it gave the odour of burning flesh. (*Literary Gazette,* March 6th, 1847.)

It appears that physicians in Egypt of the most opposite opinions on the general question of contagion, are agreed with regard to the non-contagion of the disease in its sporadic form. Between June 1835 and December 1838, 649 cases of sporadic plague were observed in Alexandria, and 646 of these did not communicate the disease to those who touched or attended on them. In 3 it was doubtful whether it did so or not. The Franks also, in the Levant, who observe strict quarantine during epidemics, adopt no precaution against the disease when only present in a sporadic form. It appears further, that in cases imported by vessels to Europe, these vessels have *always* departed from infected places while *an epidemic was raging*. It is desirable that these researches should be carried on, and—

"If the results should confirm those already obtained, we shall be able without inconvenience to confine quarantine precautions against the importation of plague to Europe to the periods when pestilential epidemics reign. This would be a great service rendered by medicine to society.

"At present we believe we should define the conclusion of this chapter thus:

"Patients affected with sporadic plague do not appear to be able to determine centres of infection sufficiently active to transmit the disease." (p. 188.)

"Is the plague more or less transmissible according to the intensity of the epidemic, its different periods, and the organic dispositions of the persons submitted to the action of the pestilential miasmata?"

The answer is in the affirmative; age, sex, state of health, and other causes, with the condition of the atmosphere, having an evident effect upon the spread of this as of all other contagious diseases.

The third part is terminated by the following observation:

"If it is not proved that the existence of a pestilential constitution in a country where the plague is imported is necessary for the transmission and propagation of the latter, still it appears certain that this imported plague cannot effect great ravages, if it does not encounter in the climate, atmosphere, and inhabitants conditions favorable to its development." (p. 196.)

The necessity for preventing importation of plague having been proved, the period under which persons arriving from suspected places must be kept under observation is to be determined by the number of days the disease may remain latent in the human body. We showed in our former article that there was every reason to believe that the period of latency never exceeded ten days. The committee go further, and state that although a fixed and absolute limit cannot be assigned to the duration of the incubation of plague—

"Still it appears demonstrated by known facts, that far from countries where the plague is endemic and beyond epidemic foci, this disease has never broken out in compromised persons after an isolation of *eight* days. The few facts that might be regarded as exceptions to this rule are all susceptible of another interpretation." (p. 199.)

In our parliamentary correspondence we find a document strongly corroborating this statement, being a return of all the persons who had been placed under observation in the lazaretto of Alexandria during four years, 1840 to 1843 inclusive. The number of families was 538, of individuals 5240, and out of this number 43 were attacked with plague, and all *before the eighth day* after the operation of *spoglio*, which consists in washing and being separated from all clothes, bedding, or anything with which they or suspected persons had been in contact. We find on summing up the table that 12 were attacked on the *third* day, 8 on the *second*, 7 on the *first*, 5 on the *fourth*, 3 on the *sixth*, 2 on the fifth, seventh, ninth, and tenth, and 1 on the eighth.

Up to 1841, the period of quarantine after *spoglio* was eleven days, but as it was proved that in those apparently attacked on the 8th, 9th, or 10th days the disease had always really shown itself before the seventh day, in 1842 the period of observation was reduced to seven days only after the operation of spoglio in the lazaretto.

These facts were known to the Academy, and those in favour of longer quarantine brought forward the objection, that the cases were arrivals from Constantinople, and consequently the disease was a milder one than that of Egypt, and it did not follow that the period of latency was the same. But this is completely erroneous; those admitted into the lazaretto of Alexandria being almost invariably the remaining members of a family in the city, one of the members of which has died of plague, or persons who have been in communication with them.

In his communication to the committee, M. Segur-Dupeyron quotes a case which lately occurred at Malta, seventeen days after the arrival of the vessel, and forty-six after her departure from Alexandria. He argues very gravely that this could not have been a case of incubation of 46 days, but that the person must have taken the disease from having opened some of his trunks with papers and clothes after his arrival in Malta. Now we happen to have seen this man, and to know that his disease was not plague, and as the case is one which shows how every apparent fact opposing principles deduced from general observation should be examined before it is received as true, we shall give a short account of this supposed instance of 17 or 46 days' incubation.

On the 17th day after the arrival of a vessel from Alexandria, a man complained of fever, and on being visited by the quarantine physician was found in a typhoid condition, with a bubo in the groin. He had had the plague in Malta, in 1813, and stated his own conviction that he was suffering from the same disease. Two physicians, who had also had great experience of the plague in the same year, agreed with him, and the fact being calculated to materially influence proposed changes in the quarantine regulations, the chief medical officers of the army and navy were called in to the assistance of the Maltese physicians. The English gentlemen had never seen plague, but had seen a great deal of syphilis and mercurial cachexia, and gave a very strong opinion that the man was suffering from the latter, although he stoutly denied it. Every precaution was taken as if the case were plague, and the case went on from bad to worse, the English and Maltese physicians still holding opposite opinions, and the man himself stoutly adhering to his original statements. The intelligent captain of the lazaretto, suspecting some concealed motive for this conduct, went to the man, and told him that the doctors were killing him, as they were giving him medicine for plague, which would certainly kill any one suffering from syphilis. This led to a confession, but the man died before it could be formally received. Nothing satisfactory resulted from the post-mortem examination, and the case would have still remained open to dispute, the opposite assertions of the man being equally unworthy of confidence, had not his wife and two persons living in the house with them soon afterwards arrived at Malta from Alexandria. All these people were examined on oath before the Board of Health, at Malta, and swore that the man had a bubo before he left Alexandria, that he was known to have associated with a prostitute, and, on leaving Alexandria, to have had both mercurial pills and mercurial ointment in his possession. So much for M. Segur-Dupeyron and his 17 days' incubation of plague.

Let us now inquire what are the practical applications of the scientific conclusions of the committee:

"The sanitary measures which should assure the security of France may be classed under five heads:

"1. Indication of the countries from which we have to fear the importation of the plague.

"2. Precautions to be taken at the departure of ships leaving suspected countries for France.

"3. Rules to observe during the passage or stoppages.

"4. Precautions at arrival in a French port.

"5. Measures to be taken in case the plague should break out in a French town." (p. 205.)

In this chapter a want of personal knowledge among the members of the committee of lazarettos, ships, and the present means of segregation and observation is evident in every page, and, what is far less excusable, their most important conclusions are not carried out in the measures they propose. Thus they conclude, not only that the plague has never been imported except by a ship with plague cases on board which had left an infected place, but also that in every such case the plague was raging *as an epidemic* in this place at the time of the ship's departure. This is not strictly correct, as M. Segur-Dupeyron has recorded nine cases in which plague had appeared after the arrival of a ship, and had not done so during the passage. But in every such case the voyage has only been from three to six days, that is, within the usual period of latency, and every one had sailed from infected places. The latter part of their conclusion is confirmed by the experience of 200 years, and certainly proves that no quarantine is necessary, except upon ships arriving from places where plague was raging at the time of departure. This is what we long since urged, and are still convinced that it is the only means of preserving Europe from the plague, without impeding commerce and communication. It does not destroy lazarettos, nor render a properly-trained sanitary body less necessary than at present, any more than peace leads to the disbanding of a standing army. Everything should be prepared for the moment of danger, but no restrictions enforced which security renders needless. The committee still keep Syria, Turkey, the Barbary States, and Morocco subjected to quarantine, whereas their own conclusion shows that this is perfectly useless when there is no plague there. Since 1837, the period when the sanitary board was established at Constantinople, the only plague cases observed there have been in the lazaretto; these are clearly imported, and did not extend beyond the lazaretto. The same fact has been observed since 1838 in Smyrna and in Greece, and Syra, the principal lazaretto of the Grecian Archipelago; the only cases of plague have occurred in the lazarettos, and have been incontestably imported. The plagues of Barbary have always been imported, and generally by vessels with pilgrims on their return from Mecca; but this only when plague was epidemic in the countries they had left. Egypt is at present under different circumstances, as sporadic cases occasionally occur; but it is clear that the state of public health in any port at the time of a ship's departure should be the guide whether quarantine should be imposed upon her arrival in Europe.

A number of precautions are recommended before a ship leaves suspected countries, but the only important alterations in existing regulations are that the bill of health should be signed by a consular physician, and should be given on the day of departure of the ship. The latter would be a great improvement, as the present plan of granting departure within six days of the date of the certificate renders it entirely useless. The physician is recommended to remark upon cleanliness and ventilation of the ship; but when a vessel is full of cargo, he must be very sharp-sighted indeed who could discover the clean or dirty state of her hold. It is recommended that a physician should be attached to each consulate, to give the certificate of the sanitary condition of the district, but this would lead to expenses, to which merchants might reasonably object, as the consuls always give their certificates upon reports furnished by the principal medical men of the place, who must be as well informed as a consular physician could be. On the whole, the recommendation has the objectionable air of a *job*. The abolition of the suspected bill of health is very properly advised; but we should object to the rule to give a clean bill when there are *only a few* sporadic cases, and a foul bill only when these are more numerous, and prefer giving a foul bill when any plague is present, a clean bill when there is none, as one of the *very few cases* might find its way on board ship while the disease was latent. Besides it is impossible to say when the sporadic disease may become epidemic, or, in the words of the report, when an epidemic is imminent. Who can say when this is to be feared? Who can draw the line between the termination of the sporadic and the commencement of the epidemic condition? How many sporadic cases can be present before the disease can be considered epidemic? The rule of the committee is altogether too vague; too much is left to the discretion of the physician, who, if a contagionist, would scarcely ever give a clean bill of health, and if a non-contagionist, never a foul one.

The precautions during the voyage consist in ventilation and a sick list to be kept by the surgeon or captain.

The periods of quarantine on arrival in Europe are regulated as follows:

"1. For ships having a physician on board, and coming from Egypt, Syria, or Turkey, with a *clean* bill of health, the quarantine shall be ten full days from *departure*, when neither the plague nor any suspected disease is manifested on board during the voyage.

"The quarantine shall be fifteen full days from *departure* when the same ships arrive with a *foul* bill of health, if neither plague nor any suspected disease is manifested on board during the voyage.

"2. For merchant ships arrived with a *clean* bill of health, not having a physician on board, a quarantine of observation of ten full days shall be prescribed from *arrival*.

"When the same ships arrive with a *foul* bill, but without either plague or any suspected disease having appeared at sea, they shall undergo a vigorous quarantine of fifteen days from date of *arrival*." (p. 217.)

All this is pitiful yielding to existing prejudices, an evident fear of applying their own deductions from authenticated facts and long experience to actual practice. To give ten full days' quarantine from time of arrival to a ship with a *clean* bill of health is an absurd contradiction of the most important of their own conclusions; and the distinctions between ships with and without a medical man are unjust if both have a clean bill of health, and no suspected disease has appeared in either. The committee show useless severity in time of safety, and we think not sufficient caution in time of danger; for in the present state of our knowledge it appears that a period at least equal to that of the longest period of latency of the disease should pass from the *arrival* of the ship and ventilation of the cargo and effects, before the crew and passengers are admitted to free pratique, when a *foul* bill has been given at the port of departure. It would be well also in every case where a suspected disease shows itself on board ship, to leave the duration of the quarantine to the discretion of the local authorities.

Neither do we approve of the mode of procedure recommended in the report, in case plague should appear in a European town, when it is recommended to "induce or even compel the greatest possible number of inhabitants to leave the town." The accounts in Dr. White's work and the facts observed in Malta, in 1813, show that isolation is the grand point, separation of the sick from the healthy and from each other. The sick and those who have been in communication with them should be removed to well-aired places in the suburbs, not the healthy and uncompromised inhabitants. The rules are, however, on the whole, good and worthy of extract.

"If the plague appears in a house, the person affected should be immediately carried to a distant and well-ventilated situation, where he can receive all the care his case requires. All the other inhabitants of the house should repair to a place pointed out by the authorities, where they may be submitted to the inspection of a physician. The evacuated house should be cleaned, washed, ventilated, purified, and remain empty at least a month.

"If several houses should be attacked, the same conduct must be pursued in each case. Further, the greatest possible number of inhabitants should be induced or compelled to leave the town, places of refuge being assigned them, and being submitted to measures of isolation necessary to prevent the propagation of the disease to the neighbouring population." (p. 229.)

This would be impracticable. Could places of refuge be built for the inhabitants of a large town at a day's notice?

"If entire towns were the seat of an epidemic of plague, these dispositions should be put in execution on a grander scale, and with a vigorous severity, but the principles would remain unchanged. It would be always necessary, on the one hand, to endeavour to make all persons not affected by the disease leave the epidemic foci; and, on the other, to isolate and disseminate the impested in elevated and well-ventilated situations, so as to prevent the formation of centres of pestilential infection." (p. 230.)

We have now gone through this Report, and need not refer to the documents on which it is founded, having already given the inferences drawn from them, and a large portion consisting of translations from the parliamentary correspondence are analysed in our former articles. From the discussions which follow, it appears that almost every member of the committee signed the report with an understanding that he was to express his dissent to certain conclusions in the general meetings of the Academy, and their speeches show that they still entertain very opposite opinions on the leading principles of the controversy. These principles, however, have been so fully discussed in the different sections of the report that we need not again refer to them, but remark that the work as a whole, report, documents, and discussions, is by far the most complete body of evidence, and the most accurate account of the natural history of plague which has ever yet appeared, being a complete analysis of the numerous published and unpublished works which have hitherto been written, always excepting those of the English writers on the disease in Malta and Corfu; still we must express our opinion that, with practical reference to the quarantine question, the field of inquiry might have been judiciously limited, more care taken in distinguishing assertions from facts, and more definite regulations framed upon the scientific conclusions. The report is highly creditable to the Academy, and particularly so to M. Prus, who drew it up, although we think he has shown more learning, industry, and research than close reasoning, or the peculiar talent of seizing and forcibly presenting the really important facts presented to him.

Before concluding we must refer to the effects produced by this report. In the Chamber of Deputies the Minister of Commerce announced that he should regulate his proposed reforms by the advice of the Academy, but he met with considerable opposition from the superior council of health, which, being composed of peers, deputies, and bankers, wished to retain the patronage of £80,000 a year the sanitary administration of quarantine costs the country. Lively debates took place in the Chamber of Deputies, and we are happy to be able to allude to the creditable position held there by M. Bouillaud, as a proof that a scientific and practical physician can also hold an honorable and useful place in the parliament of his country. M. Thiers took part in one of the debates, and in the course of his speech made one of those sarcastic allusions to physicians, which vain pretenders have drawn upon their order, "I do not come here to talk medicine," said M. Thiers, "but common sense." The reply of M. Bouillaud was a model of tact, wit, and good taste. After summing up the argumentative part of the question, he continued—

"I am sorry to be obliged to trouble you a little longer. I never could have believed that M. Thiers, who justly prides himself upon his common sense, could have said that this was not a question of medicine, but of common sense. Gentlemen, on this point I will relate you a little anecdote which recurs to my memory. (Hear, hear.)

"Hannibal—(loud laughter)—Hannibal, during his exile, was invited to attend the course of an illustrious professor who lectured on the art of war—he had never made war, and yet he lectured on war. When Hannibal had heard the illustrious professor, he gave, on retiring, to the persons who asked his opinion on the superiority of this great professor, an answer which M. Thiers knows, and which I need not recall to his memory. (Loud laughter and cheers.)" (Journal des Débats, 4 Juin, 1846.)

To understand this joke our readers should be aware that Hannibal is a name given to M. Thiers by some of the Parisian journals, in allusion to his very warlike writings, and his very *un*warlike conduct in flying from Paris during the three days' revolution of 1830, which he had mainly contributed to excite, and his return to reap the rewards after the fight was over.

To return, however, to the results of the debate and the recommendation of the Academy. The quarantine from Constantinople has been reduced to twelve days, including the voyage in case of clean bill of health, or if the voyage exceeds twelve days, three days only of observation after arrival. Five days after arrival is imposed upon ships from Tunis, and nine upon those from Tripoli. So far Marseilles has yielded to the movement, and Malta and other Mediterranean ports have followed her example. The important effect of even this change can be understood when we state that in one day, at Marseilles, 150 vessels were admitted to pratique, on the issue of the new regulations, and an almost equal number at Malta. Egypt, however, is still exposed to a long quarantine, even when clean bills of health are issued. All these half measures, the useless quarantine upon the Barbary States, its partial removal from Turkey, its continuance upon Egypt when healthy, are the results of the timidity of the Academy in putting their scientific conclusions into practical application,—and we have still to urge our old propositions.

1. To furnish all ships with *clean* bills of health where no plague is present at the port of departure, and with *foul* bills in the opposite state.

2. To receive all ships with clean bills of health in free pratique.

3. To impose eight clear days after arrival and ventilation of cargo to all ships with a foul bill.

4. In case of any suspected disease on board, the quarantine to be regulated in duration by the local authorities.

These are plain and definite applications of science to practice, which afford security to the public and but little inconvenience to individuals, or impediment to commerce, and concentrate the attention of the sanitary guardians upon the real seat of danger. A far nobler work still remains to be accomplished—the annihilation of the scourge which has been so fatal to our race. The work is begun, and we earnestly hope that the Arab may have cause to bless our country for its completion.

P.S. Since the preceding article, which was intended for our April number, was written, we are happy to say that both the French and English governments have adopted the rational system of quarantine advocated in it and in our former articles on the subject. The boon thus conferred on commerce is immense, while it is gratifying to know that it is exclusively to the exertions of medical men that the important changes have been brought about. We give the French ordonnance, dated 18th April, in an abridged form; the shorter English document we print in full.

### I. FRENCH REGULATIONS.

"1. Vessels arriving from impested places will in future only be distinguished by *clean* and *foul* bills of health, the latter only to be given when, in a place of departure or in places therewith in free communication, some pestilential epidemic exists under circumstances dangerous to the public health, and is to be given the day of or the day before the departure of the vessel. 2. Vessels from Turkey or Egypt, with an authorized sanitary officer on board, will be admitted on arrival with a clean bill of health to free pratique if the voyage have occupied ten days. 3. Those from Turkey without a sanitary officer to have a quarantine of three days in the Mediterranean ports and one day in other French ports. 4. Those from Syria and Egypt, with a clean bill, and without a sanitary officer, to five days. 5. Those with a *foul* bill to have ten full days from arrival. 6. Those with a clean bill from Tunis to be admitted in free pratique. 7. Merchandise with a clean bill to be admitted in free pratique after a voyage of ten days, and with foul bill, three days after arrival. 8. In cases of disease on board ship the local authorities to decide the period of quarantine. 9. French sanitary physicians will be established in the Levant to sign certificates at the port of departure."

### II. ENGLISH REGULATIONS.

"Council Office, Whitehall, 18th May, 1847.

"Sir,—I am directed by the Lords of the Council to state to you for the information of the Commissioners of Customs, that their Lordships, taking into consideration the healthy state of the Levant (with reference to plague) for several years past, have come to the determination of abolishing for the present all quarantine upon vessels arriving from the Levant (Turkey, Egypt, and Syria included), whatever may be the nature of their cargo, provided such vessels are furnished with clean bills of health, having been first visited by the quarantine or other proper officer of Customs, and that the crews and all persons on board have been free from any suspicion of infectious disease during the voyage. You will therefore move the Commissioners of Customs to give the necessary instructions for carrying the above regulation into effect. I am, sir, your obedient servant,

"Charles Scovell, Esq. (Signed) W. L. BATHURST.

"Custom House, London, 21st May, 1847.

"The aforegoing copy of a letter from Mr. Bathurst (one of the Clerks of the Council in waiting) is transmitted to the collector and controller at for their information and government. By order of the Commissioners."

---

